# Letter to the editor: “Implementation of ultrasound-guided cannulation training for foundation doctors”

**DOI:** 10.1016/j.clinme.2025.100284

**Published:** 2025-01-14

**Authors:** Kushal Krishna Banerjee, Rabia Batool Hussain

**Affiliations:** Barts and the London School of Medicine and Dentistry, Garrod Building, Turner St, London E1 2AD, England

*Dear* e*ditor,*

We enjoyed reading the interesting study by Pham *et al*^1^ on implementing ultrasound-guided peripheral intravenous cannulation (US-PIVC) training for newly qualified doctors. This study highlights the potential of ultrasound in reducing failure rates associated with PIVC. This is a crucial skill for all doctors who have recently graduated. However, although the study pronounces the use of US-PIVC into the foundation curriculum, we noted some concerns that may impact the strength of the findings.

Firstly, the study relies on self-reported confidence scores as the primary outcome measure. Extensive literature indicates a weak correlation between confidence and actual competence in clinical skills​. Therefore, without objective assessments such as supervised procedural evaluations, it remains vague whether increased confidence results in reduced failure rates. Objective measures could have strengthened the evidence, thus offering a more reliable measure of clinical competence.

Finally, the authors advocate for US-PIVC integration in foundation curriculum, they acknowledge the risk of dependency on ultrasound, where dependency may ‘de-skill’ foundational PIVC techniques. This could be addressed by introducing a hybrid training model, where US-PIVC and traditional landmark-based techniques are combined. For example, competency training can include simulation scenarios without ultrasound to ensure proficiency in both techniques.

Furthermore, future research may involve randomised controlled trials. These trials may compare outcomes for resident doctors using hybrid training compared to ultrasound-only models. Therefore, this trial can assess procedural competence, failure rates and adaptability to different clinical scenarios. This can provide valuable evidence to enhance the foundation curricula and support wider competency-based training that improves confidence and clinical skills.

In conclusion, the study highlights the potential of US-PIVC training but underscores the need for larger and multicentre studies. Objective skill assessments can aid its integration into foundation curricula planning. This approach could ensure a competency-driven training that addresses both confidence and clinical skills.

## Funding

This research did not receive any specific grant from funding agencies in the public, commercial, or not-for-profit sectors.

## CRediT authorship contribution statement

**Kushal Krishna Banerjee:** Writing – review & editing, Writing – original draft. **Rabia Batool Hussain:** Writing – review & editing, Writing – original draft.

## Declaration of competing interest

The authors declare that they have no known competing financial interests or personal relationships that could have appeared to influence the work reported in this paper.
